# Glycerol carbonate as green solvent for pretreatment of sugarcane bagasse

**DOI:** 10.1186/1754-6834-6-153

**Published:** 2013-10-24

**Authors:** Zhanying Zhang, Darryn W Rackemann, William O S Doherty, Ian M O’Hara

**Affiliations:** 1 Syngenta Centre for Sugarcane Biofuels Development, Queensland University of Technology, GPO Box 2432, 2 George St, Brisbane, QLD 4001, Australia; 2 Centre for Tropical Crops and Biocommodities, Queensland University of Technology, GPO Box 2432, 2 George St, Brisbane, QLD 4001, Australia

**Keywords:** Pretreatment, Glycerol carbonate, Ethylene carbonate, Sugarcane bagasse, Microcrystalline cellulose, Enzymatic hydrolysis, Adsorption

## Abstract

**Background:**

Pretreatment of lignocellulosic biomass is a prerequisite for effective saccharification to produce fermentable sugars. In this study, “green” solvent systems based on acidified mixtures of glycerol carbonate (GC) and glycerol were used to treat sugarcane bagasse and the roles of each solvent in deconstructing biomass were determined.

**Results:**

Pretreatment of sugarcane bagasse at 90°C for only 30 min with acidified GC produced a solid residue having a glucan digestibility of 90% and a glucose yield of 80%, which were significantly higher than a glucan digestibility of 16% and a glucose yield of 15% obtained for bagasse pretreated with acidified ethylene carbonate (EC). Biomass compositional analyses showed that GC pretreatment removed more lignin than EC pretreatment (84% vs 54%). Scanning electron microscopy (SEM) showed that fluffy and size-reduced fibres were produced from GC pretreatment whereas EC pretreatment produced compact particles of reduced size. The maximal glucan digestibility and glucose yield of GC/glycerol systems were about 7% lower than those of EC/ethylene glycol (EG) systems. Replacing up to 50 wt% of GC with glycerol did not negatively affect glucan digestibility and glucose yield. The results from pretreatment of microcrystalline cellulose (MCC) showed that (1) pretreatment with acidified alkylene glycol (AG) alone increased enzymatic digestibility compared to pretreatments with acidified alkylene carbonate (AC) alone and acidified mixtures of AC and AG, (2) pretreatment with acidified GC alone slightly increased, but with acidified EC alone significantly decreased, enzymatic digestibility compared to untreated MCC, and (3) there was a good positive linear correlation of enzymatic digestibility of treated and untreated MCC samples with congo red (CR) adsorption capacity.

**Conclusions:**

Acidified GC alone was a more effective solvent for pretreatment of sugarcane bagasse than acidified EC alone. The higher glucose yield obtained with GC-pretreated bagasse is possibly due to the presence of one hydroxyl group in the GC molecular structure, resulting in more significant biomass delignification and defibrillation, though both solvent pretreatments reduced bagasse particles to a similar extent. The maximum glucan digestibility of GC/glycerol systems was less than that of EC/EG systems, which is likely attributed to glycerol being less effective than EG in biomass delignification and defibrillation. Acidified AC/AG solvent systems were more effective for pretreatment of lignin-containing biomass than MCC.

## Background

Lignocellulosic biomass is the most abundant renewable resource on earth and has the potential to partly replace fossil-based resources for production of fuels and chemicals. Lignocellulosic biomass consists of three major components, cellulose, hemicellulose and lignin with cellulose being embedded in a matrix of the latter two structural biopolymers. Pretreatment is essential to improve cellulose accessibility to cellulase enzymes for production of fermentable sugars [1,2]. However, the major obstacle to using lignocellulosic biomass is the high processing costs, which are mainly associated with pretreatment reactor capital costs and consumption of energy and chemicals used for pretreatment [1-3].

Pretreatment of lignocellulosic biomass at low temperatures of ≤ 100°C can save up to 50% energy consumption compared to alternative pretreatments (*e.g.*, dilute acid pretreatments with water as solvent) operated at temperatures of 160 – 180°C [3]. While water is the most benign, environmentally friendly and importantly cheap solvent, it provides limited impact on biomass deconstruction under mild pretreatment temperatures of ≤ 100°C unless used in conjunction with concentrated mineral acids [4,5]. However, the use of large amounts of acid introduce issues regarding reactor corrosion and acid recovery and requires the treatment of the acid residue, producing a lot of wastes [6].

Low temperature pretreatment processes with the use of high boiling point solvents such as some ionic liquids do not require high pressure reactors and reduce the rates of reactor corrosion allowing less expensive materials to be used for reactor construction (*e.g.*, thinner reactor walls and lower priced alloys). A few imidazolium ionic liquid-based pretreatments have been used to achieve glucan digestibilities of ≥ 90% for lignocellulosics pretreated at temperatures of ≤ 130°C [7,8]. However, the high solvent costs of these ionic liquids could hamper their applications at industrial scales. Recently, pretreatment of rice straw with low cost and renewable chlolinium amino acid ionic liquid-water mixtures at a temperature of 90°C have also been reported [9]. However, this pretreatment requires a reaction time of 12 h to achieve sugar yields of > 80%.

We have previously reported a low temperature (90°C) process for atmospheric pretreatment of sugarcane bagasse with mixtures of ethylene carbonate (EC) and ethylene glycol (EG), which are industrially available, low cost solvents [10]. The EC/EG-based pretreatment produces biomass having a maximal glucan enzymatic digestibility of 93%, making it very effective. The high effectiveness of the EC/EG-based systems is attributed to (1) EC’s ability to reduce particle size (length) due to its high static relative dielectric constant (*ϵ*), (2) EG’s ability to defibrillate biomass and (3) both solvents’ ability to remove lignin from biomass under acidified conditions. Although EC itself is considered a solvent of low toxicity [11], its decomposition product, EG (also used in the pretreatment system) is toxic to human health. Long term exposure to EG may cause metabolic acidosis, cardiopulmonary failure and acute renal failure [12]. We are therefore interested in other cyclic carbonates, which have similar or higher *ϵ* values but are “greener” and less toxic than EC.

**Figure 1:**
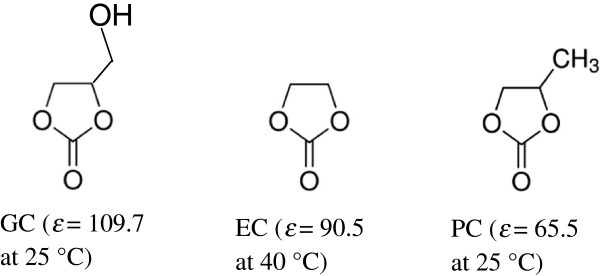
**Molecular structures and *ϵ *** **values of GC, EC and PC **[13]**.**

Glycerol carbonate (GC) is such a cyclic alkylene carbonate (AC). GC has a similar structure to propylene carbonate (PC) (with one hydrogen from the methyl group in PC replaced by a hydroxyl group) (Figure 1) and has the highest *ϵ* value among these three cyclic carbonates [13]. GC is classified as a low toxicity, sustainable solvent and is a promising versatile building block chemical with numerous applications [11]. Both GC and its decomposition product glycerol show very low toxicities [11]. Interestingly, GC can be synthesized by reaction of CO_2_[14,15], urea [16,17] and dialkyl carbonates [18-22] with glycerol in the presence of chemical or enzymatic catalysts. In particular, glycerol is produced in large quantities in the biodiesel industry, making it readily available and cheap. GC synthesis is being suggested as a way to valorize glycerol from the biodiesel process [11]. Furthermore, GC has a boiling point of 354°C, much higher than that of EC (260°C). Its decomposition product glycerol has a boiling point of 290°C, which is much higher than EC’s decomposition product EG (197°C). The higher boiling points of GC and glycerol make them more suitable for atmospheric reaction than the EC/EG systems.

We also hypothesized that the presence of one hydroxyl group in GC’s molecular structure may enhance biomass swelling and defibrillation as occurs with alkylene glycols (AGs). We herein investigated the effectiveness of GC/glycerol systems to deconstruct sugarcane bagasse for enzymatic saccharification in comparison to the EC/EG systems. Furthermore, both GC/glycerol and EC/EG systems were used to pretreat microcrystalline cellulose (MCC) such that the effect of the solvent solely on cellulose could be examined. Enzymatic digestion of and congo red (CR) adsorption on MCC pretreated with these solvent systems were analysed and compared to better understand the roles of the individual solvents in pretreatment.

## Results and discussion

### Pretreatment of sugarcane bagasse

#### *Biomass composition and component recovery*

Pretreatments were conducted with AC/AG solvents containing 1.2% H_2_SO_4_. With this acid concentration, pretreatment by EC/EG was most effective at 90°C for 30 min in terms of delignification, xylan removal and glucan digestibility [10]. Table 1 shows the results of biomass composition and component recovery. All the GC/glycerol pretreatments improved glucan content in biomass but decreased xylan and lignin contents. Decreasing GC content in the solvent decreased glucan content but increased xylan and lignin contents. The highest glucan content (75.6 wt%) in biomass was achieved with pretreatment by GC alone which also resulted in the lowest xylan (7.6 wt%) and lignin (8.6 wt%) contents. The biomass yield (recovery) decreased with decreasing glycerol content because of increased removal of xylan and lignin. Glucan recovery remained high (≥ 90%) at all GC:glycerol ratios.

**Table 1:** Biomass composition, component recovery, glucan digestibility and glucose yield of pretreated bagasse

**Solvent type**	**AC:AG**	**Content in solid residue (wt%)**			**Component recovery (%)**				**72 h glucan digestibility (%)**	**Total glucose yield (%)**
		**Glucan**	**Xylan**	**Lignin**	**Biomass**	**Glucan**	**Xylan**	**Lignin**		
Untreated bagasse		43.8±1.3	20.2±0.4	27.5±0.6	100.0	100.0	100.0	100.0	12.0±0.3	12.0±0.3
GC:glycerol	1:0	75.6±0.7	7.6±0.1	8.6±0.2	51.9±1.8	89.6±1.0	19.5±0.5	16.3±0.6	89.9±1.7	80.5±2.1
	9:1	74.8±0.2	9.9±0.0	9.7±0.4	53.9±0.8	92.0±1.0	26.3±0.7	19.0±0.7	90.2±2.2	83.0±3.3
	4:1	74.9±0.1	10.2±0.0	9.1±0.2	55.6±1.6	95.1±1.1	28.0±0.6	18.4±0.4	87.1±2.1	82.9±0.9
	2:1	72.0±0.4	11.9±0.1	11.3±0.1	57.6±2.3	94.7±2.7	34.0±0.2	23.7±0.9	86.9±1.8	82.3±1.2
	1:1	68.7±0.2	12.3±0.0	12.0±0.1	59.5±1.0	93.3±1.4	36.3±0.8	25.9±0.3	87.6±1.7	81.7±1.8
	0:1	56.6±0.0	13.8±0.0	23.4±0.0	73.6±1.9	95.1±0.6	50.3±0.8	62.5±0.5	61.3±2.2	58.3±2.2
EC:EG	1:0	64.2±1.2	7.0±0.1	20.4±0.5	61.8±2.5	90.6±0.6	21.3±0.4	45.8±1.2	16.3±1.3	14.8±1.6
	4:1	76.7±0.3	10.1±0.0	7.5±0.3	53.1±0.5	93.0±1.2	26.5±0.3	14.4±0.3	97.1±1.8	90.3±2.1
	0:1	67.2±0.8	14.1±0.2	13.4±0.3	62.4±0.9	95.7±0.8	43.6±1.1	30.6±1.0	74.7±2.8	71.5±1.2

Compared to GC/glycerol pretreatments, pretreatment by EC alone removed significantly less lignin but slightly more xylan. As a result, glucan content after EC pretreatment was ~11% lower than that after GC pretreatment. Both EC and GC pretreatments led to lower glucan recoveries compared to the pretreatments with mixed carbonate and glycol solvents. This was likely attributed to the high solution acidity (due to the high solvent dielectric constants), which resulted in hydrolysis of more cellulose components. In the previous study, it was found that the optimal ratio of EC:EG for EC/EG systems was 4:1 [10]. At this ratio, EC/EG pretreatment led to slightly higher delignification than pretreatments by GC/glycerol systems with GC:glycerol ratios from 1:0 to 4:1. EG pretreatment removed more lignin than glycerol pretreatment possibly due to EG’s high lignin solubility [23].

#### *Biomass characterisation*

**Figure 2:**
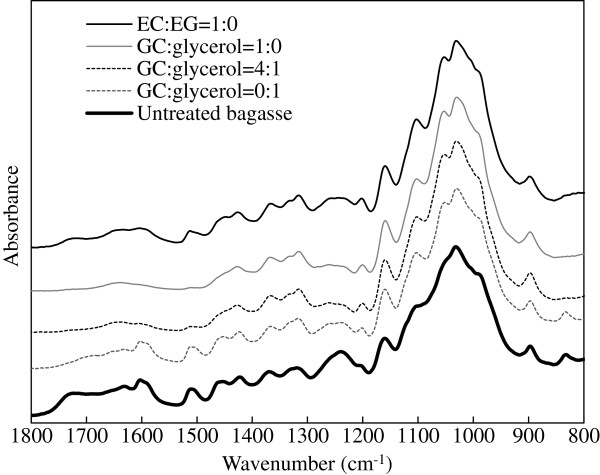
FTIR spectra of pretreated bagasse samples.

Samples were also characterised using Fourier transform infrared spectroscopy (FTIR), X-ray powder diffraction (XRD) and scanning electron microscopy (SEM). Figure 2 shows the FTIR spectra of biomass samples. The intensities of lignin-associated peaks at ~1732 cm^-1^ (related to the uronic acid ester bonds formed between the carboxylic acid group in hemicellulose and the phenolic hydroxyl group in lignin, and/or between the carboxylic acid group from lignin hydroxycinnamic acid and the hydroxyl group from arabinofuranose unit [24,25]), at 1605 cm^-1^ and 1515 cm^-1^ (assigned to aromatic skeleton vibrations in lignin [26]), at 1460 cm^-1^ (possibly associated with the methoxy group in lignin [27]), at 1240 cm^-1^ (assigned to β-ether bonds in lignin [26]) and at 835 cm^-1^ (which belongs to a C-H out of plane vibration in lignin [28]) diminished or disappeared with bagasse pretreated with GC solutions (Figure 2). Some of these peaks associated with lignin were also weaker for bagasse pretreated with GC compared to bagasse pretreated with EC, and so explain the difference in biomass yield. The results imply that, under similar conditions, GC provides a better delignification capacity than EC.

The region of 1200–1000 cm^-1^ represents C-O stretch and deformation bands in cellulose, lignin and residual hemicellulose [29]. The increase in band intensity at 1200 cm^-1^ of pretreated bagasse may be related to the increase in the proportion of the glucan content. The band intensity at 1105 cm^-1^, which corresponds to crystalline cellulose [30] increased in all the pretreated bagasse samples compared to the untreated bagasse, indicating that the pretreatment removed amorphous components in the bagasse. XRD analysis also showed that the pretreatments did not decrease cellulose crystallinity (Additional file 1: Figure S1) and the estimated CrIs of the pretreated biomass (0.73 - 0.75) were slightly higher than that of untreated bagasse (0.68). Slight increase in CrI was also observed in our previous studies where acidified solvents were used to pretreat sugarcane bagasse due to the removal of amorphous components [10,31]. Figure 2 also shows that the peak at 1050 cm^-1^, which is associated with the C-O stretch in cellulose and hemicellulose [28], was prominent in pretreated bagasse, indicating the increase in glucan content. The peak at 898 cm^-1^ is characteristic of β-glycosidic linkages between the sugar units in carbohydrates [26].

As shown by the scanning electron microscopy (SEM) images, the average particle width of untreated bagasse was ~250 – 500 μm while after GC pretreatment the width was reduced to ~40 – 150 μm (Additional file 2: Figure S2a), similar to, if not smaller, than the particle width range of fibres from EC pretreatment (Additional file 2: Figure S2b). The fibres from GC pretreatment seem fluffy compared to the compact nature of the fibres obtained EC pretreatment (Additional file 2: Figures S2a and S2b). So defibrillation as well as size reduction occurred with GC pretreatment. Pretreatment by glycerol reduced biomass particle width to 60 – 120 μm (Additional file 2: Figure S2c) while pretreatment by EG partially defibrillated biomass fibres (with a width range of 20 – 30 μm) (Additional file 2: Figure S2d).

#### *Enzymatic hydrolysis of pretreated bagasse*

Table 1 also shows the glucan digestibility and glucose yield obtained from saccharification experiments. It is worth mentioning that without acid catalyst, AC/AG pretreatments had little effect on glucan digestibility of pretreated bagasse (data not shown). After 72 h enzymatic hydrolysis, cellobiose was not detected. Pretreatment with mixtures of GC/glycerol led to glucan digestibilities of 87 – 90% and glucose yields of 82 – 83%, ~25% higher than those with glycerol alone. The glucan digestibility and glucose yield obtained with GC pretreatment alone were 90% and 80% respectively, only slightly lower than that with mixtures of GC/glycerol. In comparison, our previous results showed that water-based pretreatment with 1.2 wt% HCl as catalyst (which has higher acidity than 1.2 wt% H_2_SO_4_ used in this study) in a sealed vessel at 130°C for 60 min only led to a glucan digestibility of only 38% [31].

These results from GC/glycerol pretreatments are significantly different from those results from EC/EG pretreatments. As previously reported the glucan digestibility and glucose yield of bagasse pretreated with the mixture of EC/EG was much higher than bagasse pretreated with EC alone and also significantly higher than bagasse pretreated with EG alone [10]. Repeated experiments with EC/EG solvents in this study confirmed our previous observation (Table 1). Pretreatment with EC alone led to a glucan digestibility of ~16% and a glucose yield of ~15%, which were significantly lower than those with pretreatment by GC alone. The higher glucose yield achieved with GC pretreatment may be attributed to GC’s better delignification (Table 1) and defibrillation abilities (fluffy biomass generated from GC pretreatment as shown in Additional file 2: Figure S2a and S2b). As fluffy particles have larger specific surface area than compact particles, the accessibility of cellulose to cellulases is improved [32-34]. It was also noted that the pretreatment effectiveness using mixtures of GC/glycerol was slightly lower than the effectiveness using mixtures of EC/EG. This is attributed to the better biomass defibrillation and delignification [35] capacity of EG compared to glycerol. When glycerol in the GC/glycerol system was replaced by EG, a glucose yield of ~90% was achieved, which was comparable to that of bagasse pretreated by EC/EG (data not shown).

The difference in pretreatment effectiveness between GC and EC is likely attributed to differences in their molecular structures (Figure 1). The presence of one hydroxyl group in an organic solvent enhances GC’s capacity to delignify biomass similar to typical alcohol and phenol solvents [36-39] explaining why GC is a better biomass delignification and defibrillation solvent than EC. In addition, *ϵ* of GC (109.7 at 25°C) is higher than that of EC (90.5, 40°C) [13]. For an acid-catalysed reaction in non-aqueous solvent, the acid potential is associated with the *ϵ* of the solvent [40]. It is generally considered that a solvent with higher *ϵ* also has a higher acid potential accounting for the similar (if not smaller) biomass particle size produced by GC compared to EC pretreatment.

#### *Pretreatment solution*

Glucose and 5-hydroxymethylfurfural (HMF, a glucose degradation product) which are generated in many acid-catalysed lignocellulose pretreatment processes, were not detected in any of the pretreatment solutions. The yields of xylose and furfural (a xylose degradation product) were very low (Table 2). The low yields of xylan derivatives may be attributed to the production of xylan oligomers [41,42] and/or the formation of glycol-glycosides (glycol-glucosides and glycol-xylosides) [10,43]. Previous studies have shown that glycol-glycosides existed in the solutions collected after pretreatment using acidified glycols [43]. Glycol-glycosides were hydrolysed to glycol and sugars upon dilution and hydrolysis of the pretreatment solution [10,43]. In this study, formation of glycosides with glycerol was also likely because of the presence of glycerol. As shown in Table 2, the xylose yield increased significantly after hydrolysis of the pretreatment solutions. Also, small amounts of glucose were present in the hydrolysed pretreatment solution. The xylose yield decreased with decreasing GC content possibly due to less xylan removed from bagasse (Table 1) and the inhibition of hydrolysis of glycerol-xylosides at higher glycerol concentrations. The same trend was observed with glucose yield. For EC/EG systems, the xylose yield in the hydrolysed pretreatment solution from EC pretreatment was lower than that in the solution from EC/EG pretreatments possibly because of the production of furfural (Table 2) and the formation of undetected xylose and furfural polymerisation or degradation products such as humins [44].

**Table 2:** Component yield before and after hydrolysis of pretreatment solution

**Solvent type**	**AC:AG**	**Pretreatment solution**		**Hydrolysed pretreatment solution**^**1**^		
		**Xylose (%)**	**Furfural (%)**	**Glucose (%)**	**Xylose (%)**	**Furfural (%)**
GC:glycerol	1:0	8.4±1.7	0.3±0.0	4.2±0.3	73.2±2.5	1.9±0.3
	9:1	6.8±0.4	0.2±0.0	2.8±0.3	71.3±3.5	2.1±0.3
	4:1	5.1±0.6	0.2±0.1	2.4±0.2	67.1±5.1	2.0±0.2
	2:1	4.4±0.1	0.1±0.1	2.0±0.1	57.1±2.7	1.0±0.2
	1:1	4.0±0.1	-	1.5±0.1	53.0±5.8	0.5±0.1
	0:1	1.5±0.7	-	0.6±0.2	30.7±4.0	0.3±0.0
EC:EG	1:0	2.6±0.5	9.5±0.5	3.1±0.2	38.8±3.7	11.7±0.8
	4:1	2.9±0.4	0.4±0.2	1.8±0.5	66.8±8.0	1.2±0.2
	0:1	1.5±0.3	-	0.5±0.1	31.6±5.7	0.6±0.1

1. Solvent solution collected after pretreatment was diluted to a water content of 75 wt% and hydrolysed in a sealed pressure tube for 30 min at 130°C.

Similar to the EC/EG system [10], pretreatment by GC/glycerol was also accompanied by the gradual decomposition of GC to glycerol. For GC pretreatment, ~6 wt% of GC was converted to glycerol after pretreatment at 90°C for 30 min (data not shown), which was slightly higher than that of EC conversion to EG (~3 wt%) under similar conditions [10]. Nevertheless, ACs are very stable at neutral pH. Therefore, after pretreatment the solvent solution can be neutralised and further processed to remove impurities (lignin with large molecular weights may be precipitated by adding water into the solution [18]; lignin with small molecular weights may be removed by adsorption with activated carbon [45]; soluble sugar-related products may be separated by chromatography techniques) and water (*e.g.*, by vacuum evaporation). The kinematic viscosity (centistokes) of GC (61 at 25°C) is much lower than that of glycerol (714 at 25°C) although it is significantly higher than water (0.9 at 25°C) [46,47], indicating that GC process is more readily amenable than glycerol process to pretreatments at high biomass loadings, to separation of pretreated biomass and to recovery of solvent.

### Pretreatment of MCC

#### *Cellulose yield and enzymatic hydrolysis*

To better understand the mechanism of pretreatment with AC/AG systems, MCC was also pretreated with GC/glycerol and EC/EG systems and the substrates were hydrolysed by cellulases. As shown in Table 3, the cellulose yields after pretreatment of MCC by either GC/glycerol or EC/EG systems were 87 – 93%, close to the glucan recoveries of pretreated bagasse, confirming that AC/AG systems does not hydrolyse glucan significantly under the present reaction conditions. The lowest cellulose yield was achieved with pretreatment with AC alone whereas the highest cellulose yield was obtained with AG alone, although the difference was not significant. In addition, the highest amounts of glucose were detected in the hydrolysed pretreatment solutions with ACs (Additional file 3: Figure S3). These data indicated that AC pretreatment increased depolymerisation of cellulose.

**Table 3:** Cellulose yield, glucan digestibility and glucose yield of pretreated MCC

**Solvent type**	**AC:AG**	**Cellulose yield (%)**	**72 h glucan digestibility (%)**	**Total glucose yield (%)**
GC:glycerol	1:0	87.0±1.4	76.0±2.9	66.1±2.5
	4:1	91.4±3.6	75.3±2.7	68.8±2.5
	0:1	93.0±0.9	83.2±1.3	77.4±1.2
EC:EG	1:0	89.2±2.1	60.1±2.0	53.6±1.8
	4:1	91.1±2.6	74.1±1.3	67.5±1.2
	0:1	92.9±2.6	81.0±0.6	75.3±0.6
Untreated MCC		100.0	72.2±0.5	72.2±0.5

Table 3 also shows the glucose yield and glucan digestibility of pretreated MCC after 72 h enzymatic hydrolysis. MCC pretreated by EC alone had the lowest glucan digestibility of only 60.0%, lower than that of untreated MCC (72.2%) and also much lower than that of MCC pretreated by GC alone (76.0%). Residual solvents were not detected in the washed biomass by HPLC analysis (data not shown), indicating the enzymatic hydrolysis of pretreated bagasse were not inhibited by the solvents. Pretreatment with EC/EG mixture (4:1) led to an increase in glucan digestibility by 14% compared to that with EC pretreatment. However, there was no obvious difference between the glucan digestibilities of MCC pretreated by GC alone and the mixture of GC/glycerol. MCC pretreated by glycerol and EG had the highest glucan digestibilities. This may be attributed to the better deconstruction of MCC by polyols. SEM images show that the particle width range of untreated MCC was ~25 – 60 μm (Additional file 4: Figure S4a). After pretreatment, the particle width range was reduced. The particle width range was ~12 – 30 μm for MCC pretreated by GC alone (Additional file 4: Figure S4c) and is similar to the particle width range for MCC pretreated by EC or glycerol alone (Additional file 4: Figures S4b and S4e). The average particle width (~18 – 40 μm) of MCC pretreated by EG alone was slightly higher than other pretreated MCC (Additional file 4: Figure S4d). However, the MCC pretreated by EG alone seemed fluffy (*i.e.,* defibrillated) compared to that pretreated by EC alone. Total glucose yield followed a similar trend to glucan digestibility as the cellulose yield only changed slightly among pretreatments.

#### *Correlation of glucan digestibility with CR adsorption*

**Figure 3:**
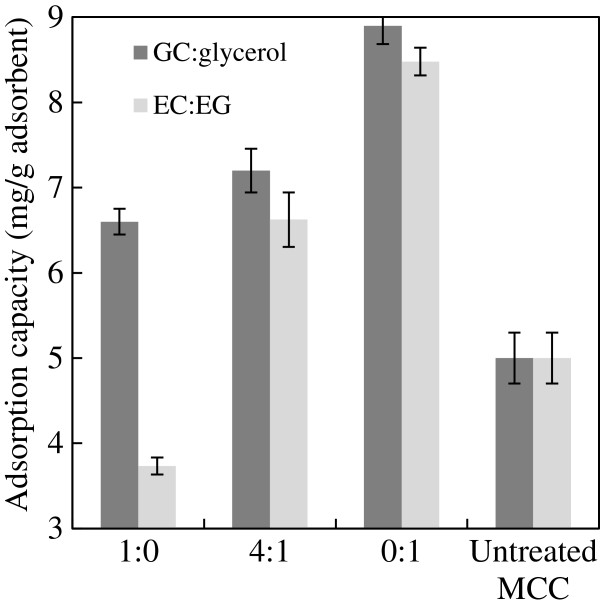
CR adsorption capacity of pretreated MCC.

**Figure 4:**
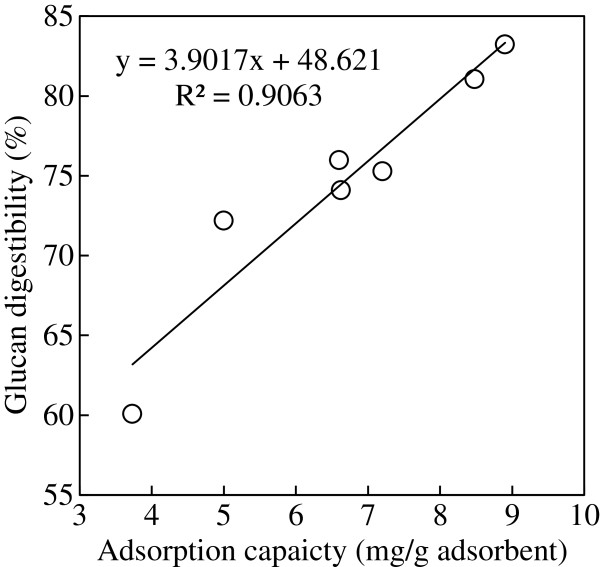
Correlation of glucan digestibility of MCC samples with CR adsorption capacity.

The effect of pretreatment on biomass surface area was evaluated by dye adsorption tests. CR adsorption on MCC matched Langmuir isotherm (Additional file 5: Figure S5), indicating there is a positive linear relationship between dye adsorption capacity and the biomass surface area [48]. As shown in Figure 3, MCC pretreated by EC alone had the lowest CR adsorption capacity while glycerol- and EG-pretreated MCC had the highest CR adsorption capacities. The high adsorption capacity of MCC pretreated by acidified EG was likely attributed to its ability to swell cellulose [49] and thus produced fluffy biomass. Although MCC pretreated with glycerol was not as fluffy as EG-pretreated MCC, pretreatment by glycerol possibly produced porous biomass [35], which also increased the biomass surface area for CR adsorption. A good linear correlation (R^2^ = 0.9063) of glucan digestibility with CR adsorption capacity was observed for the pretreated MCC (Figure 4).

## Conclusions

Pretreatment of sugarcane bagasse with acidified GC alone was much more effective that the pretreatment with acidified EC alone. Up to 50 wt% of GC could be replaced by glycerol without having a negative effect on the pretreatment effectiveness. The maximum glucan digestibility of GC/glycerol systems was less than that of EC/EG systems, which is likely attributed to glycerol being less effective than EG in biomass delignification and defibrillation. The results also showed that AC/AG solvent systems were more effective for pretreatment of lignin-containing biomass than MCC.

Although GC and EC are considered non-toxic to human health, EG is much more toxic than glycerol. From the aspect of operational safety, GC/glycerol systems may be preferred over EC/EG systems. Currently, the price of GC is high as it is not produced commercially. Development of technology for GC synthesis based on the low cost glycerol and CO_2_ feedstocks may decrease GC production cost and make the use of GC for processing biomass more competitive.

## Methods

### Materials

Sugarcane bagasse was collected from Racecourse Sugar Mill (Mackay Sugar Limited) in Mackay, Australia. Sugarcane bagasse was washed with hot water at 90°C to remove residual sugars to a negligible amount. The washed sugarcane bagasse was air-dried, and gently shaked on a sieve having an aperture size of 1.0 cm to remove pith and the residues were ground to fine particles by a cutter grinder (Retsch® SM100, Retsch GmBH, Germany). The milled bagasse was screened and particles having width range of 250 – 500 μm were collected and stored for pretreatment. The moisture of the sieved bagasse particles was 7.1 wt%. Bagasse particles mainly consisted of 43.8 wt% glucan, 20.2 wt% xylan, 3.3 wt% arabinan, 27.5 wt% lignin, 2.5 wt% acetyl and 2.1 wt% ash. GC, glycerol, EC, EG, MCC (Avicel® PH-101) and CR were purchased from Sigma-Aldrich (US). Accellerase™ 1000 (Batch no. 1600877126), a Danisco product (Genencor Division, Danisco Inc., US), was purchased through Enzymes Solutions Pty. Ltd (Australia). Accellerase™ 1000 contained 30.4 mg protein/mL enzyme solution, which was measured using Bradford Protein Assay Kit purchased from Bio-Rad (US). The filter paper activity of Accellerase™ 1000 was ~40 FPU/mL, which was measured using a method developed by the National Renewable Energy Laboratory (NREL, US) [50]. All the chemicals used in this study were analytic standard reagents.

### Pretreatment experiment

The pretreatment solvent (4.90 g) was transferred into a 60 mL pressure tube (10.2 cm (length) × 3.81 cm (diameter), Ace Glass Inc., USA) which was immersed in a silicone oil bath preheated to 95°C. The pressure tube was not sealed and pretreatment was conducted at atmospheric pressure. The heating element was equipped with a magnetic stirring device with a stirring speed of 300 rpm (Ika Labortechnik, Germany). A picture of this pretreatment system was shown in Additional file 6: Figure S6. The pressure tube containing solvent was preheated for about 5 min to reach 90°C (measured by an external thermometer) and 33 μL of 98 wt% H_2_SO_4_ was added and the solution mixed for 30 s. Thereafter, 0.538 g of bagasse (0.5 g of dry biomass) or 0.5 g of MCC was transferred into the pressure tube. The ratio of total liquid to sugarcane bagasse (dry weight) was 10:1 (w/w) (AC/AG solvents to bagasse = 9.8:1, w/w). After 30 min of reaction time, 5 mL of water was added to the pressure tube and the mixture was thoroughly mixed. The mixture was filtered (Whatman 541 filter paper) to collect the pretreated biomass. The filtrate was collected and frozen for further analysis. The pretreated bagasse was washed with 200 mL distilled water (2 × 100 mL/wash). The washed pretreated bagasse was further washed with 50 mM NaOH solution (2 × 20 mL/wash) followed by further water wash (2 × 100 mL/wash). The washed pretreated bagasse was collected. Pretreated MCC was only washed with water (2 × 100 mL/wash). Half of the filtered biomass was freeze-dried for moisture analysis and stored for further analyses (SEM, XRD and biomass compositional analysis), while the other half of the filtered biomass was stored at 4°C for enzymatic hydrolysis. All the pretreatments were conducted in triplicate.

### Enzymatic hydrolysis

Enzymatic hydrolysis was carried out in a 20 mL glass vial containing 5 g solution in which half of the pretreated and washed biomass (equivalent to 0.130 – 0.250 g dry biomass due to the difference in biomass yields) was added. The reaction solution contained 0.05 M citrate buffer to maintain pH 4.8 and 0.02 wt% sodium azide to prevent the growth of microorganisms. The dosage of Accellerase for enzymatic hydrolysis was 0.025 mL Accellerase/g solution (45–50 FPU/g glucan due to the difference in glucan recovery). The reaction was carried out at 50°C for 72 h in a rotary incubator (Ratek OM 11 Orbital Mixer, Australia) with shaking speed of 150 rpm. After 72 h enzymatic hydrolysis, 0.5 mL of solution was withdrawn and then centrifuged at 9,000 *g* for 5 min. 0.1 mL supernatant was diluted 10 times by de-ionized water. The diluted sample was filtered through 0.22 μm disk filter prior to sugar analysis by a high performance liquid chromatography (HPLC) system. All the enzymatic hydrolysis experiments were conducted in triplicate.

### Characterisation of biomass samples

Biomass samples were characterised by FTIR, SEM, XRD and compositional analysis. FTIR spectra of the samples were recorded between 4000 cm^-1^ and 500 cm^-1^ using a Thermo Nicolet Nexus 870 system (Thermo Nicolet, USA) with the processing software Omnic 7.3. SEM was used to record the surface morphological features of bagasse before and after pretreatment. The samples were coated with gold using a Leica EMS CD 005 system prior to analysis by FEI scanning electron microscope (Quanta 200 3D, USA).

The X-ray diffractometer (PANalytical, Netherlands) with Cu K_α_ radiation (*λ* = 1.5418 nm) was operated at a voltage of 40 kV and a current of 40 mA. The 2*θ* range was from 4° to 40° in steps of 0.033° at a rate of 2.6°/min. Crystallinity index (CrI) was calculated by:

$$\mathrm{CrI}=\frac{I_{002}-I_{\mathrm{am}}}{I_{002}}$$

where *I*_*002*_ at 2*θ* = 21.5 – 23.0° is the total intensity of crystalline and amorphous components, *I*_*am*_ at 2*θ* = 17 – 19° is the “valley” intensity of amorphous cellulose, hemicellulose and lignin considering the shift of these peaks after pretreatment [10]. After XRD analysis, the biomass was recovered for compositional analysis using a modified method, which was based on a standard method developed by the NREL [51], however, instead of using 300 mg of biomass sample, 100 mg of each sample was used for analysis (due to the limited size of samples). The acid and water amounts added were also reduced proportionately. All the other operational procedures were the same as the standard method.

To better understand the effect of pretreatment on biomass properties, dye adsorption studies to reveal surface area change of MCC after pretreatment were conducted. A stock CR solution of 600 mg/L was prepared and the pH was adjusted to 7.0 by adding dilute NaOH or HCl solution Dye adsorption experiment was conducted at room temperature (24°C) in a 20 mL glass bottle with 10 mL CR solution (200 mg/L) and 5 g/L pretreated MCC. Adsorption was carried out at 24°C for 20 h in a rotary incubator (Ratek OM 11 Orbital Mixer, Australia) with shaking speed of 150 rpm. After 20 h adsorption, the optical density of the CR solution was monitored at 497 nm and the concentration was calculated using a standard calibration curve.

### HPLC analysis

A HPLC system with a Bio-Rad Aminex HPX-87H column and Waters refractive index detector was used to detect and quantify sugar derivatives such as 5-hydroxymethylfurfural (HMF) and furfural in the pretreatment solution. The mobile phase was 5 mM H_2_SO_4_ at a flow rate of 0.6 mL/min. The column temperature was 65°C. A Phenominex RPM monosaccharide column was used to determine the sugars generated from pretreatment solutions and enzymatic hydrolysis. The pretreatment solution was neutralised with CaCO_3_ prior to sugar and solvent analysis. The column temperature was 85°C and the mobile phase was water at a flow rate of 0.5 mL/min.

### Calculations

Biomass yield was calculated based on the following equation:

(1) $$\begin{array}{l} \text{Biomass}\;\mathrm{yield}\\ \;=\frac{\text{Dry}\;\text{biomass}\;\text{weight}\;\text{after}\;\text{pretreatment}\times 100\%}{\text{Dry}\;\text{weight}\;\text{of}\;\text{untreated}\;\text{biomass}} \end{array}$$

For MCC, the biomass yield was the same as cellulose yield.

Component (glucan, xylan and lignin) recovery in pretreated bagasse was calculated as follows:

(2) $$\begin{array}{l} \text{Component}\;\text{recovery}\\ \;=\frac{\text{Component}\;\text{content}\;\text{in}\;\text{pretreated}\;\text{biomass}\times \text{biomass}\;\text{yield}\times 100\%}{\text{Total}\;\text{component}\;\text{in}\;\text{untreated}\;\text{bagasse}} \end{array}$$

Glucose enzymatic hydrolysis yield of pretreated biomass was calculated based on the following equation:

(3) $$\begin{array}{l} \text{Glucose}\;\text{enzymatic}\;\text{hydrolosis}\;\text{yield}\\ \;=\frac{\text{Total}\;\text{glucose}\;\text{in}\;\text{enzymatic}\times 162/180\times 100\%}{\text{Total}\;\text{glucan}\;\text{in}\;\text{untreated}\;\text{biomass}} \end{array}$$

where 162 is the molecular weight (MW) of glucose unit in glucan and 180 is the MW of glucose.

Glucan enzymatic digestibility of pretreated biomass was calculated based on the following equation:

(4) $$\begin{array}{l} \text{Glucan}\;\text{enzymatic}\;\text{digestability}\\ \;=\frac{\text{Glucose}\;\text{enzymatic}\;\text{hydrolosis}\;\text{yeild}\times 100\%}{\text{Glucan}\;\text{recovery}} \end{array}$$

The yields of glucose (HMF, xylose and furfural) detected in pretreatment solution on total glucan (xylan) in untreated biomass was calculated based on the following equations:

(5) $$\begin{array}{l} \text{Glucose}\;\text{yield}\\ \;=\frac{\text{Total}\;\text{glucose}\;\text{in}\;\text{pretreatment}\;\text{solution}\times 162/180\times 100\%}{\text{Total}\;\text{glucan}\;\text{in}\;\text{untreated}\;\text{biomass}} \end{array}$$

where 162 is the MW of glucose unit in glucan and 180 is the MW of glucose.

(6) $$\begin{array}{l} \text{Xylose}\;\text{yield}\\ \;=\frac{\text{Total}\;\text{xylose}\;\text{in}\;\text{pretreatment}\;\text{solution}\times 132/150\times 100\%}{\text{Total}\;\text{xylan}\;\text{in}\;\text{untreated}\;\text{biomass}} \end{array}$$

where 132 is the MW of xylose unit in xylan and 150 is the MW of xylose.

(7) $$\begin{array}{l} \text{HMF}\;\text{yield}\\ \;=\frac{\text{Total}\;\text{HMF}\;\text{in}\;\text{pretreatment}\;\text{solution}\times 162/126\times 100\%}{\text{Total}\;\text{glucan}\;\text{in}\;\text{untreated}\;\text{biomass}} \end{array}$$

where 162 is the MW of glucose unit in glucan and 126 is the MW of HMF.

(8) $$\begin{array}{l} \text{Furfural}\;\text{yield}\\ \;=\frac{\text{Total}\;\text{furfural}\;\text{in}\;\text{pretreatment}\;\text{solution}\times 132/96\times 100\%}{\text{Total}\;\text{xylan}\;\text{and}\;\text{arabinan}\;\text{in}\;\text{untreated}\;\text{biomass}} \end{array}$$

where 132 is the MW of xylose unit in xylan and 96 is the MW of furfural.

The extent of GC decomposition (the yield of glycerol) after pretreatment by GC alone was calculated based on the following equation:

(9) $$\begin{array}{l} \mathrm{GC}\;\text{decomposition}\\ \;=\frac{\text{Total}\;\text{glycerol}\;\text{in}\;\text{pretreatment}\;\text{solution}\times 118/92\times 100\%}{\text{Total}\;\text{GC}\;\text{in}\;\text{initial}\;\text{pretreatment}\;\text{solution}} \end{array}$$

where 118 is the MW of GC and 92 is the MW of glycerol.

CR adsorption capacity (mg/g MCC) was calculated based on the follow equation:

(10) $$\begin{array}{l} \text{Dye}\;\text{adsorption}\;\text{capacity}\\ \;=\frac{\text{Total}\;\text{dye}‒\text{free}\;\text{dye}\;\text{in}\;\text{solution}\;\text{after}\;\text{adsorption}}{\text{Total}\;\text{MCC}\;\text{in}\;\text{solution}} \end{array}$$

All the data shown in this study are the means of triplicate experiments with standard deviation also shown.

## Abbreviations

AC: Alkylene carbonate; AG: Alkylene glycol; CR: Congo red; EC: Ethylene carbonate; EG: Ethylene glycol; ϵ: Static relative dielectric constant; FTIR: Fourier transform infrared spectroscopy; GC: Glycerol carbonate; HMF: 5-hydroxymethylfurfural; HPLC: High performance liquid chromatography; MW: Molecular weight; PC: Propylene carbonate; PG: Propylene glycol; SEM: Scanning electron microscopy.

## Competing interests

The authors declare that they have no competing interests.

## Authors’ contributions

ZZ designed and conducted the experiments, analysed and discussed the results, and prepared the manuscript. DR, WD and IO participated in discussion of the results and provided suggestions on preparation and revision of the manuscript. All authors read and approved the final manuscript.

## Supplementary Material

Additional file 1: Figure S1 XRD spectra of representative samples. Click here for file

Additional file 2: Figure S2 SEM images of bagasse samples pretreated by (a) GC, (b) EC, (c) glycerol and (d) EG. Click here for file

Additional file 3: Figure S3 Glucose yield in hydrolysed pretreatment solution after MCC pretreatment (Pretreatment solution was diluted to a water content of 75 wt% and incubated in a sealed pressure tube for 30 min at 130°C). Click here for file

Additional file 4: Figure S4 SEM images of (a) untreated MCC, MCC samples pretreated by (b) EC, (c) GC, (d) EG and (e) glycerol. Click here for file

Additional file 5: Figure S5 Langmuir isotherm of CR adsorption on MCC. Click here for file

Additional file 6: Figure S6 Picture of the reactor system. Click here for file
