# Supplementary figures and images for: Glycerol carbonate as green solvent for pretreatment of sugarcane bagasse
Source: Biotechnol Biofuels. 2013 Oct 24;6:153. doi: 10.1186/1754-6834-6-153 (PMC4015548; doi:10.1186/1754-6834-6-153)

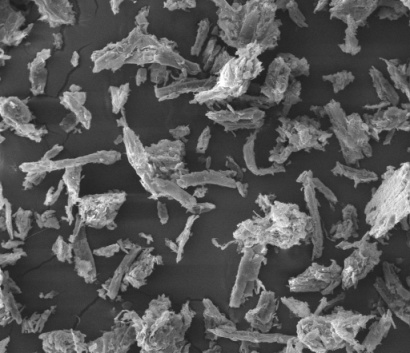


**a**

**200 μm**


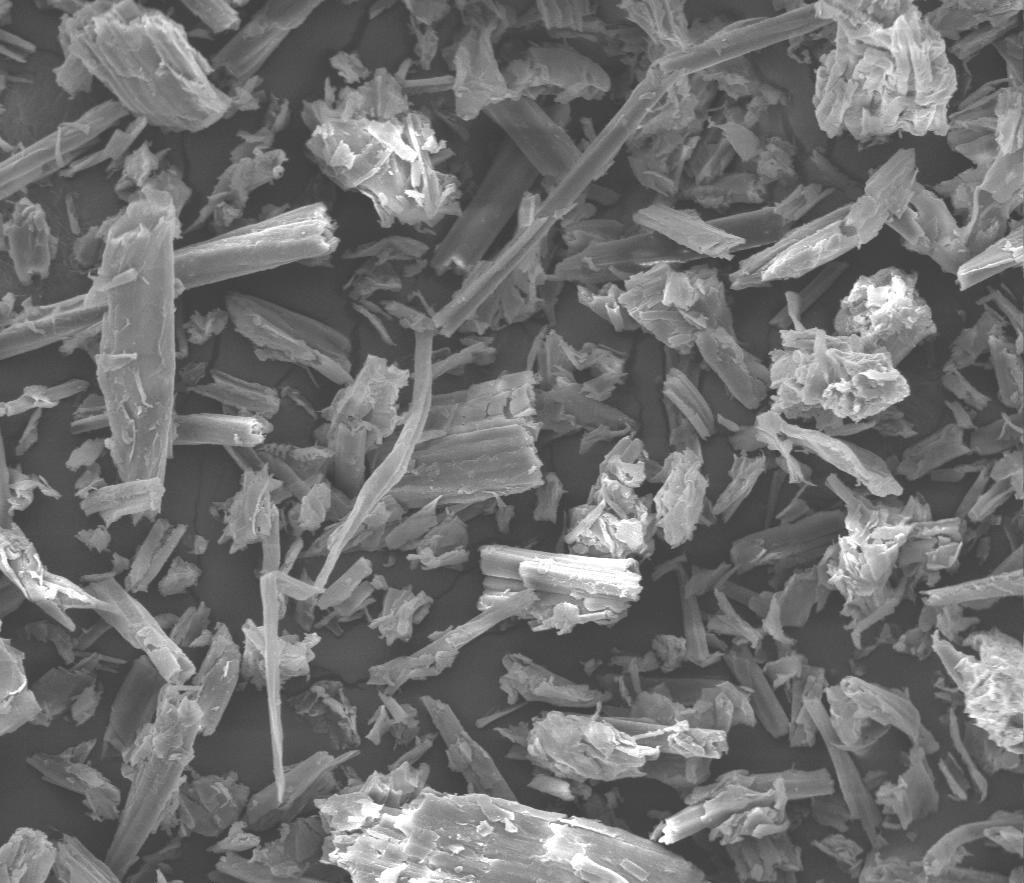


**b**

**200 μm**


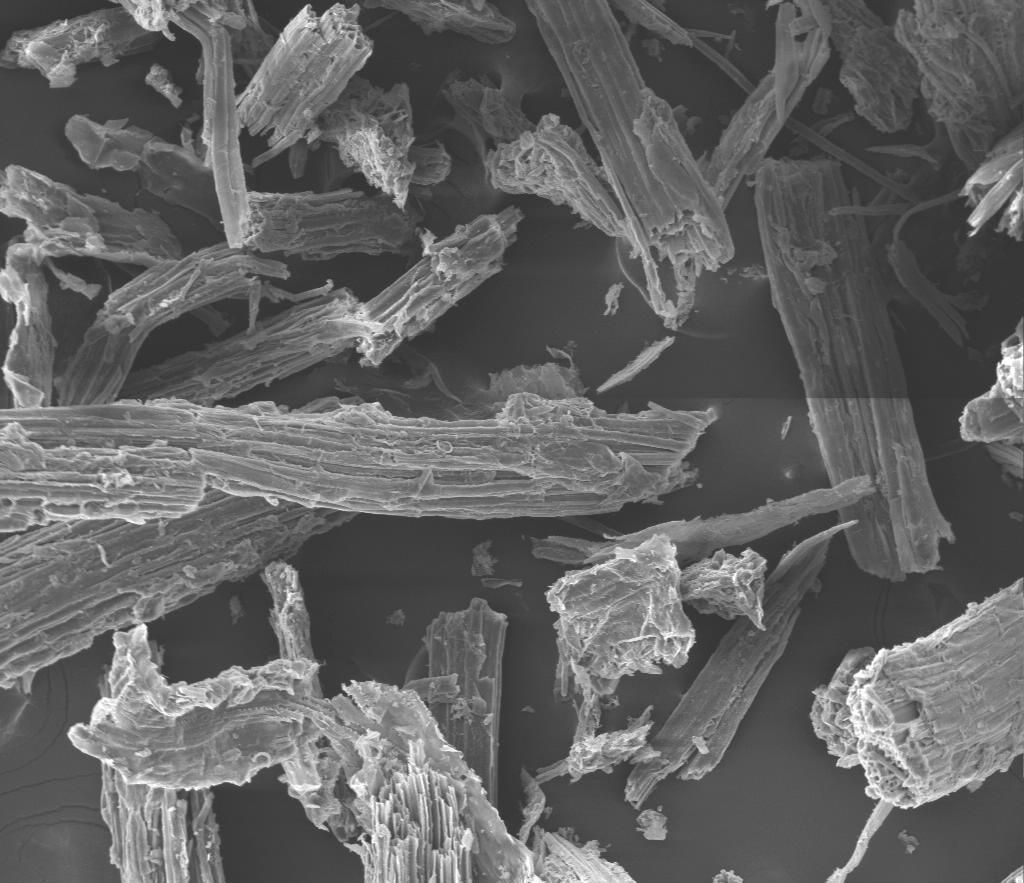


**c**

**500 μm**


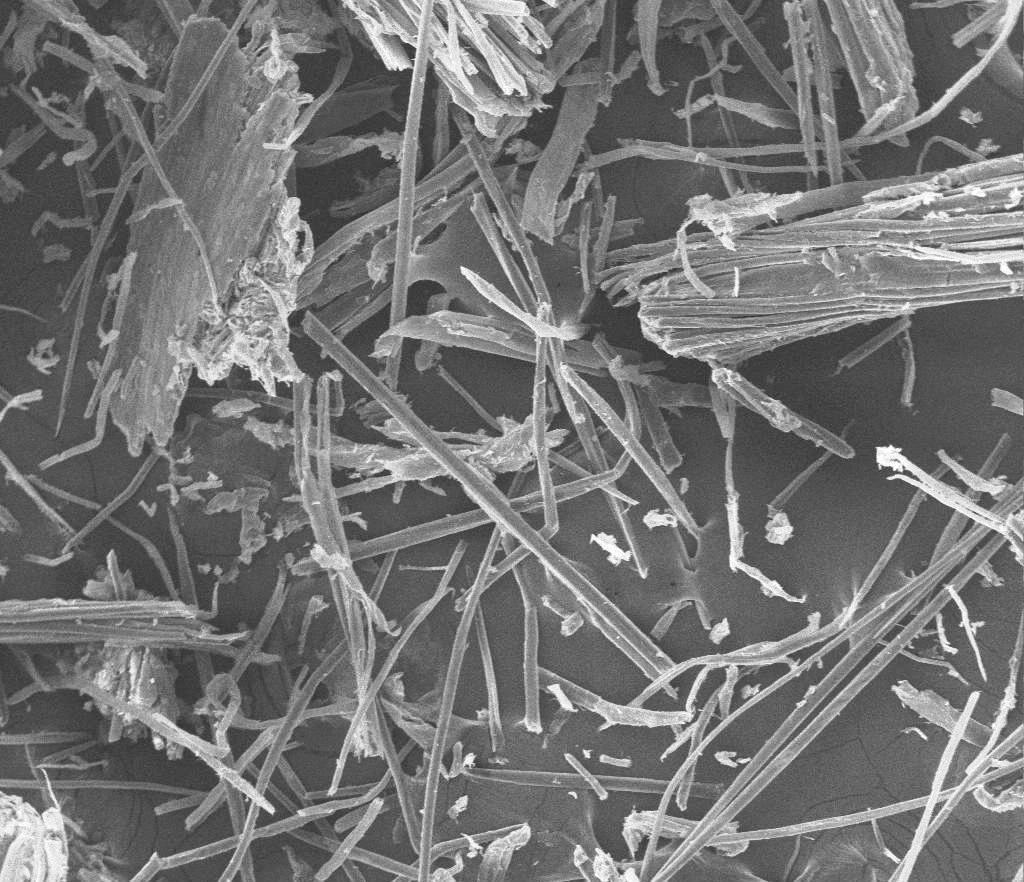


**d**

**500 μm**

Supplement: Additional file 2: Figure S2 — SEM images of bagasse samples pretreated by (a) GC, (b) EC, (c) glycerol and (d) EG. [file 1754-6834-6-153-S2.docx]

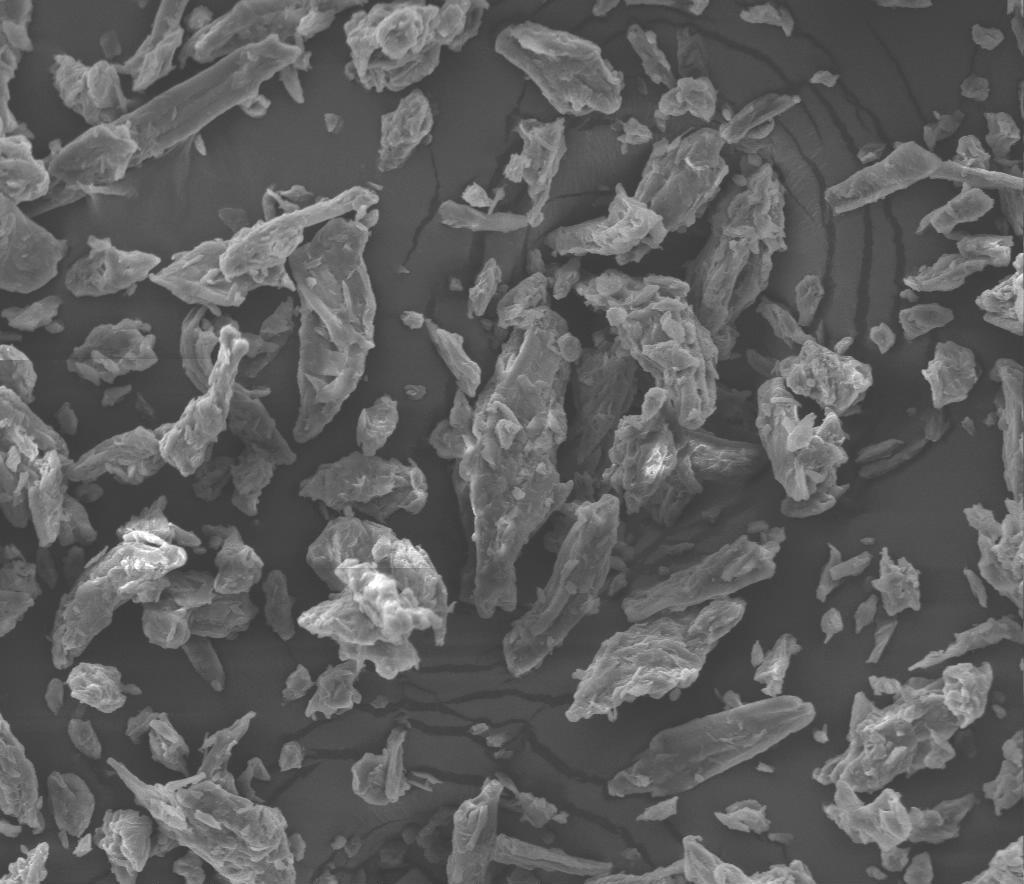


**a**

**200 μm**


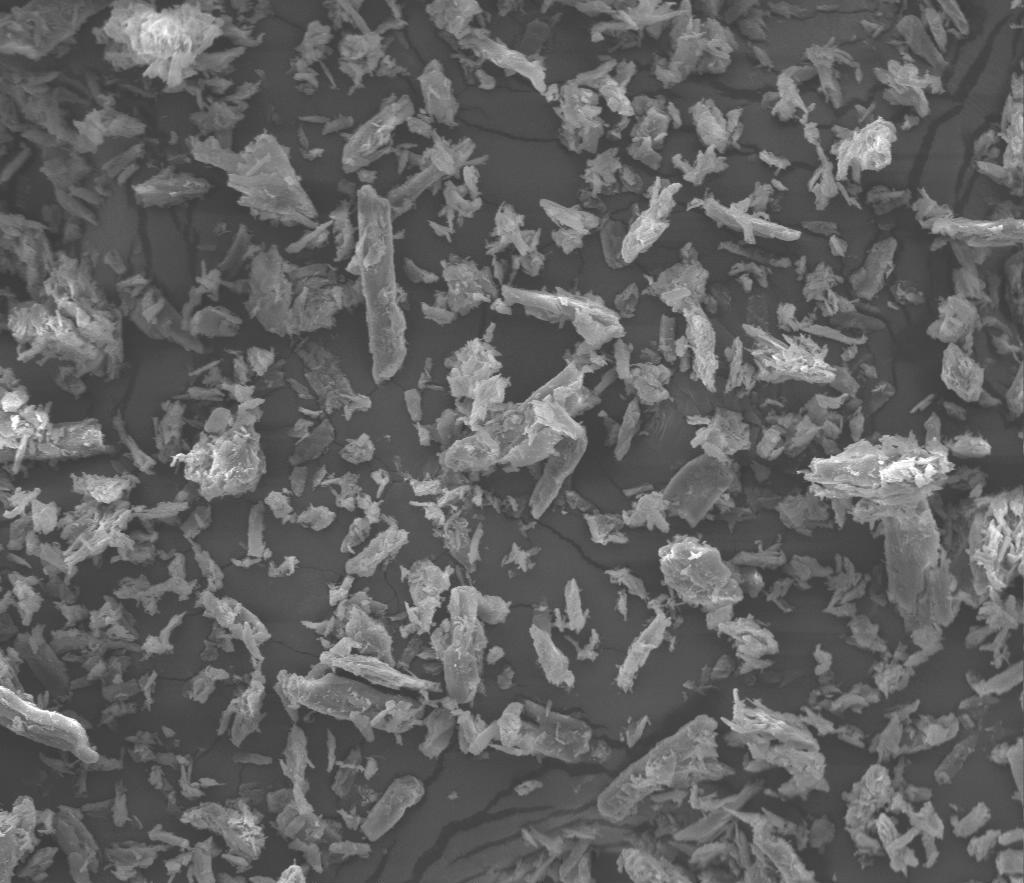


**b**

**200 μm**


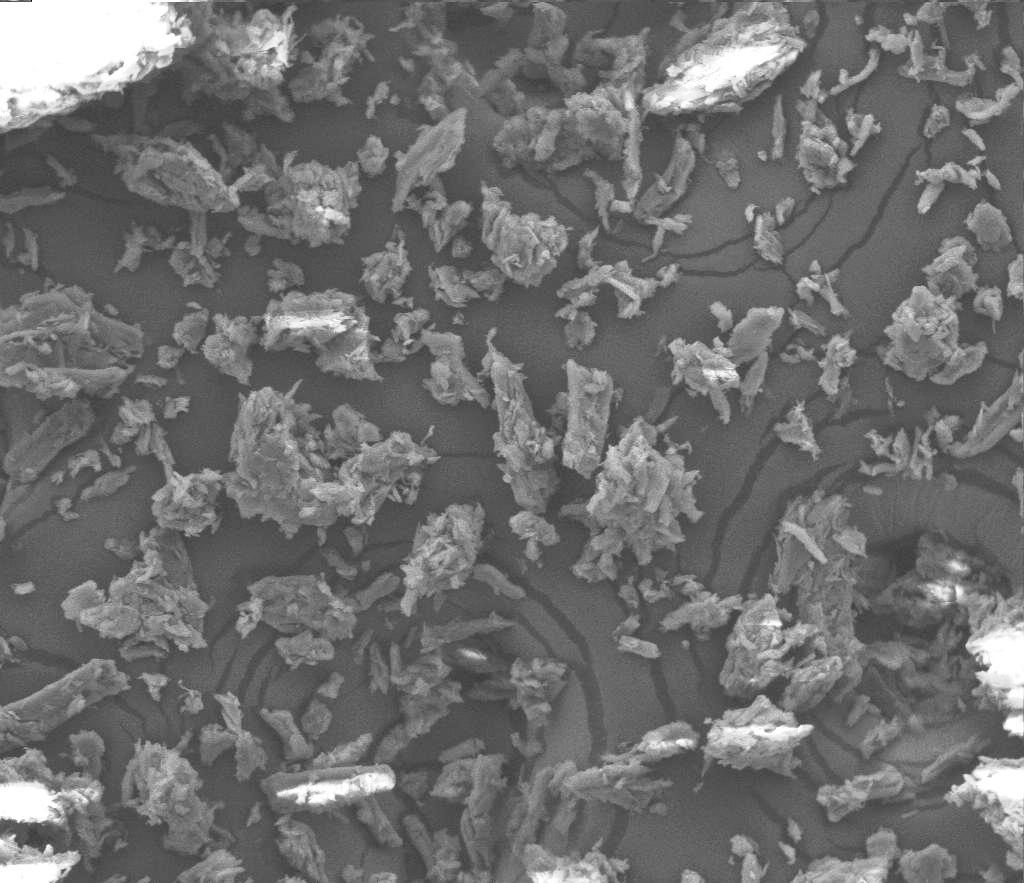


**c**

**200 μm**


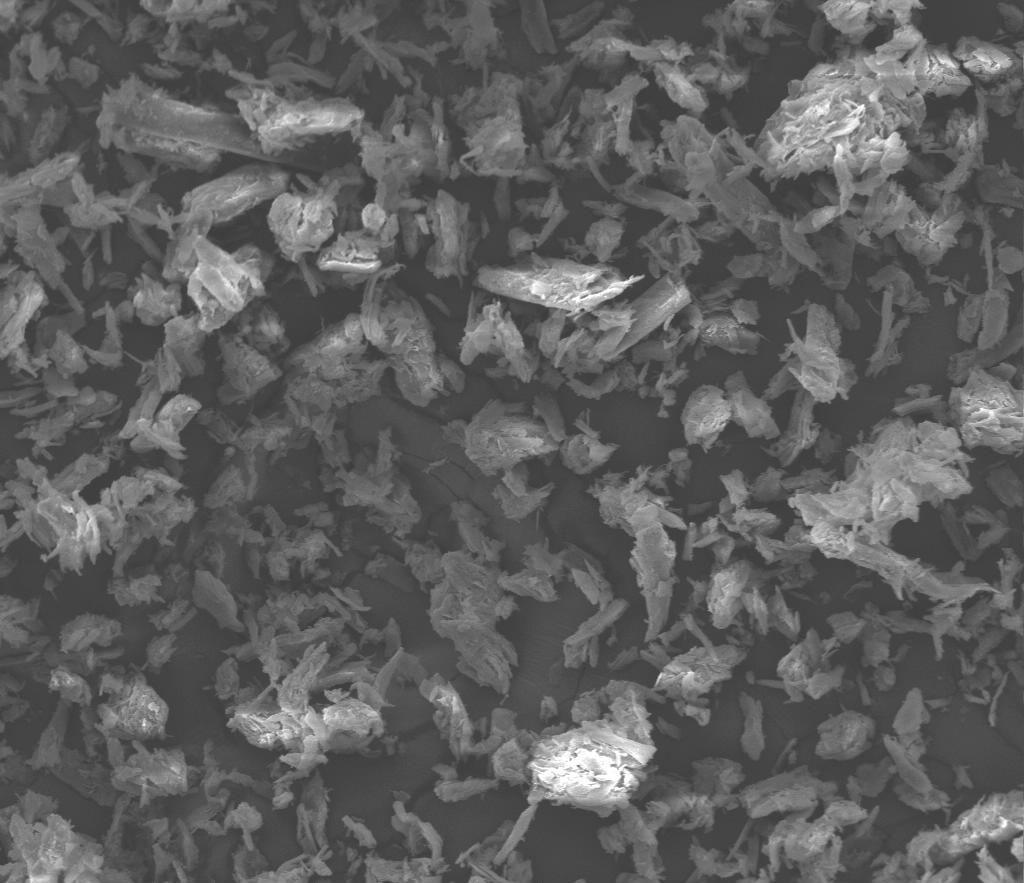


**d**

**200 μm**


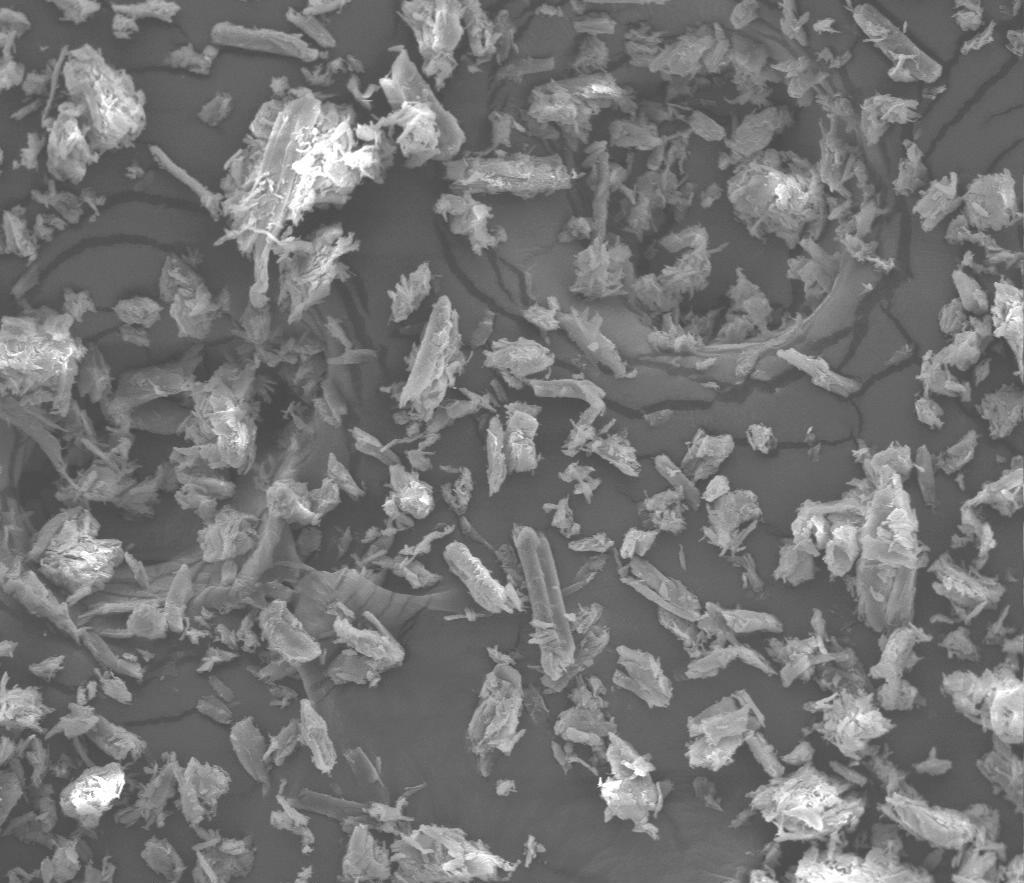


**e**

**200 μm**

Supplement: Additional file 4: Figure S4 — SEM images of (a) untreated MCC, MCC samples pretreated by (b) EC, (c) GC, (d) EG and (e) glycerol. [file 1754-6834-6-153-S4.docx]
