# Supplementary material for: Glycerol carbonate as green solvent for pretreatment of sugarcane bagasse
Source: Biotechnol Biofuels. 2013 Oct 24;6:153. doi: 10.1186/1754-6834-6-153 (PMC4015548; doi:10.1186/1754-6834-6-153)
Supplement: Additional file 5: Figure S5 — Langmuir isotherm of CR adsorption on MCC. [file 1754-6834-6-153-S5.docx]

Batch adsorption was conducted in a 20 mL glass vial at room temperature (24 °C). Adsorption was carried out for 20 h to reach equilibrium. The glass bottle contained 10 mL CR solution and 5 g/L MCC. Glass vials were agitated at 150 rpm in an orbital incubator (OM11, Ratek, Australia). Initial dye concentrations in the solutions were 50, 100, 200, 300, 400, 500 and 600 mg/L respectively for determination of adsorption isotherm. The adsorption experiment was conducted in triplicate.

More details on adsorption and determination of isotherm type refer to (i) Zhang et al., Chemical Engineering Journal, 2011, v178, p122-128, and (ii) Zhang et al., Industrial Crops and Products, 2013, v42, p41-49.
