# Supplementary material for: Glycerol carbonate as green solvent for pretreatment of sugarcane bagasse
Source: Biotechnol Biofuels. 2013 Oct 24;6:153. doi: 10.1186/1754-6834-6-153 (PMC4015548; doi:10.1186/1754-6834-6-153)
Supplement: Additional file 6: Figure S6 — Picture of the reactor system. [file 1754-6834-6-153-S6.docx]

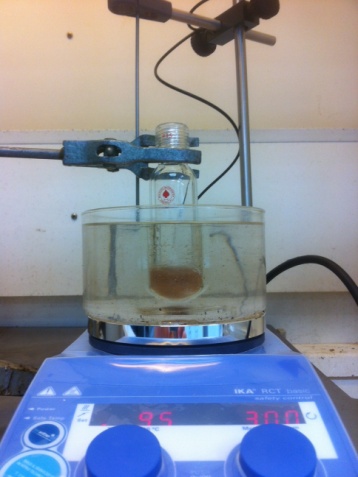


1

2

4

3

6

7

5

1. Oil bath thermometer, 2. Oil bath, 3. Hot plate, 4. Oil bath set-up temperature, 5. Open pressure tube, 7. Reaction solution, 8. Set-up stirring speed. The oil bath set-up temperature was 95 °C, which resulted in a temperature of 90 °C in the reaction solution (measured by an external thermometer).
