# Supplementary material for: Glycerol carbonate as green solvent for pretreatment of sugarcane bagasse
Source: Biotechnol Biofuels. 2013 Oct 24;6:153. doi: 10.1186/1754-6834-6-153 (PMC4015548; doi:10.1186/1754-6834-6-153)
Supplement: Supplementary file 4 [file 1754-6834-6-153-S1.docx]

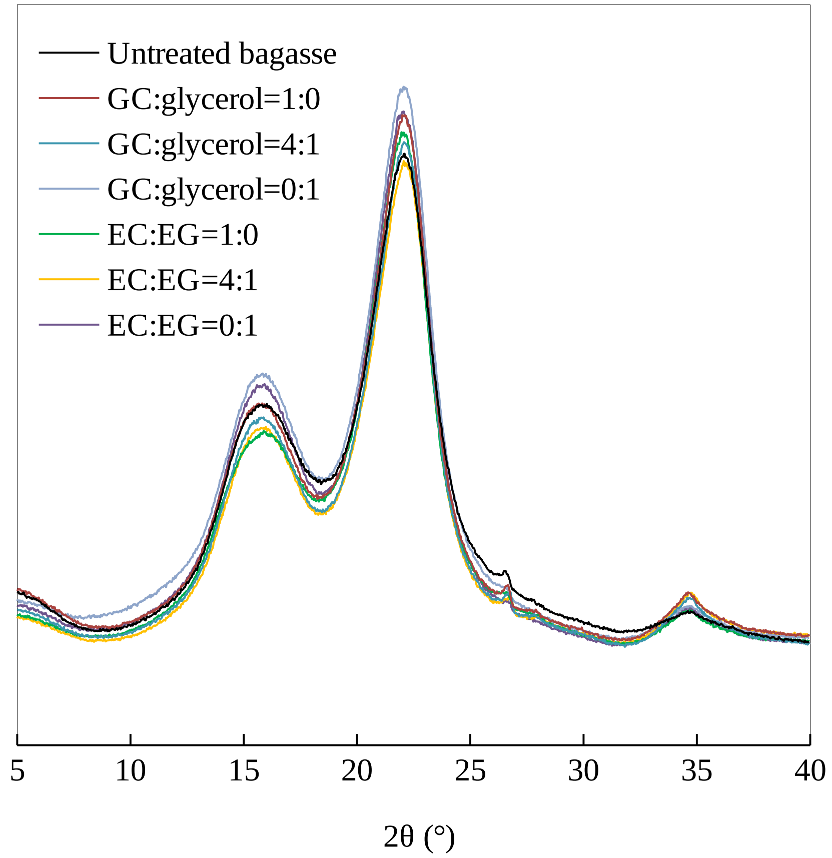
Untreated bagasse
GC:glycerol=1:0
GC:glycerol=4:1
GC:glycerol=0:1
EC:EG=1:0
EC:EG=4:1
EC:EG=0:1
5
10
15
20
25
30
35
40
2θ (°)
