# Supplementary material for: Glycerol carbonate as green solvent for pretreatment of sugarcane bagasse
Source: Biotechnol Biofuels. 2013 Oct 24;6:153. doi: 10.1186/1754-6834-6-153 (PMC4015548; doi:10.1186/1754-6834-6-153)
Supplement: Supplementary file 5 [file 1754-6834-6-153-S3.docx]

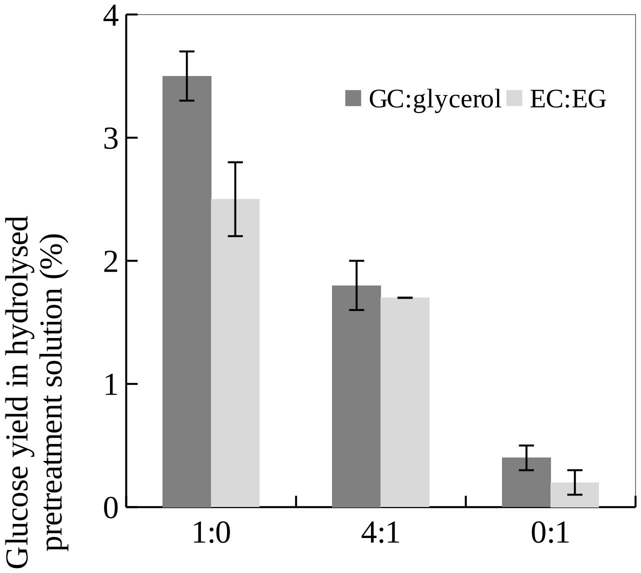

GC:glycerol
EC:EG
Glucose yield in hydrolysed pretreatment solution (%)
0
1
2
3
4
1:0
4:1
0:1
